# Nationwide seroepidemiology of canine exposure to *Ehrlichia canis* and *Babesia* spp. in Portugal (2015–2025): One Health biosentinel surveillance

**DOI:** 10.1186/s13071-026-07522-x

**Published:** 2026-06-22

**Authors:** Ricardo Lopes, Lima de Carvalho, Vanessa Silva, Cátia Fernandes, Ana Patrícia Lopes, Elsa Leclerc Duarte, Luís Cardoso, Ana Cláudia Coelho

**Affiliations:** 1 CEDIVET Veterinary Laboratories, Lionesa Business Hub, Leça do Balio, Portugal; 2 Department of Veterinary Sciences, University of Trás-os-Montes e Alto Douro (UTAD), Vila Real, Portugal; 3 Department of Veterinary and Animal Sciences, University Institute of Health Sciences (IUCS), CESPU, Gandra, Portugal; 4 AniCura Santa Marinha Veterinary Hospital, Vila Nova de Gaia, Portugal; 5 Animal and Veterinary Research Centre (CECAV), Associate Laboratory for Animal and Veterinary Sciences (AL4AnimalS), UTAD, Vila Real, Portugal; 6 Department of Veterinary Medicine, School of Science and Technology, University of Évora, Évora, Portugal; 7 Mediterranean Institute for Agriculture, Environment and Development (MED), Global Change and Sustainability Institute (CHANGE), University of Évora, Évora, Portugal

**Keywords:** *Babesia* spp., Biosentinels, Canine seropositivity, *Ehrlichiacanis*, One Health, Tick-borne infections, Vector-borne diseases, Zoonosis

## Abstract

**Background:**

*Ehrlichia canis* and canine *Babesia* spp. are widely distributed tick-borne pathogens of veterinary and One Health relevance. Contemporary nationwide data on canine exposure and associated determinants in Portugal are limited. This study aimed to estimate seropositivity proportions and explore epidemiological determinants of exposure to *E. canis* and *Babesia* spp. in clinically suspected dogs from Portugal over an 11-year period (2015–2025).

**Methods:**

Serum samples from 2960 dogs collected between 2015 and 2025 were analysed using commercial enzyme-linked immunosorbent assay kits to detect immunoglobulin G antibodies to *E. canis* and *Babesia* spp. Associations with demographic and geographic variables were assessed using chi-square and Fisher’s exact tests. Univariable and multivariable logistic regression were used to identify potential risk factors (*P* < 0.05), and model performance was evaluated using the Hosmer–Lemeshow test, Nagelkerke’s R^2^, and the area under the receiver operating characteristic curve.

**Results:**

Overall seropositivity was 4.0% (116/2,872) for *E. canis* and 12.0% (337/2,818) for *Babesia* spp. Borderline results accounted for 3.0% (88/2,960) and 4.8% (142/2,960), respectively. *E. canis* seropositivity varied significantly by Nomenclature of Territorial Units for Statistics level 2 region (*P* < 0.001), with highest values in the Algarve (15.3%; odds ratio [OR] = 4.3, *P* < 0.001) and Setúbal Peninsula (50.0%; OR = 23.9, *P* = 0.025). Age was associated with *E. canis* exposure (*P* = 0.014), with increased odds in dogs aged 6 to < 11 years and ≥ 11 years (both OR = 2.1, *P* < 0.05). No significant geographical association was observed for *Babesia* spp. (*P* = 0.803), and sex and age were not significant predictors. Seropositivity to *Babesia* spp. was a risk factor for seropositivity to *E. canis* (OR = 3.6, *P* < 0.001). Multivariable models retained no independent predictors (Nagelkerke R^2^ = 0.15; area under the curve = 0.71).

**Conclusions:**

This study provides a nationwide, long-term retrospective assessment of serological exposure to *E. canis* and *Babesia* spp. among clinically suspect dogs in Portugal. Exposure to *E. canis* showed marked regional heterogeneity, particularly in southern Portugal, whereas *Babesia* spp. showed no significant regional association. Co-seropositivity, although uncommon, is consistent with shared tick-borne exposure pathways. These findings support risk-based tick control and continued surveillance within a One Health framework.

**Graphical Abstract:**

**Figure:**
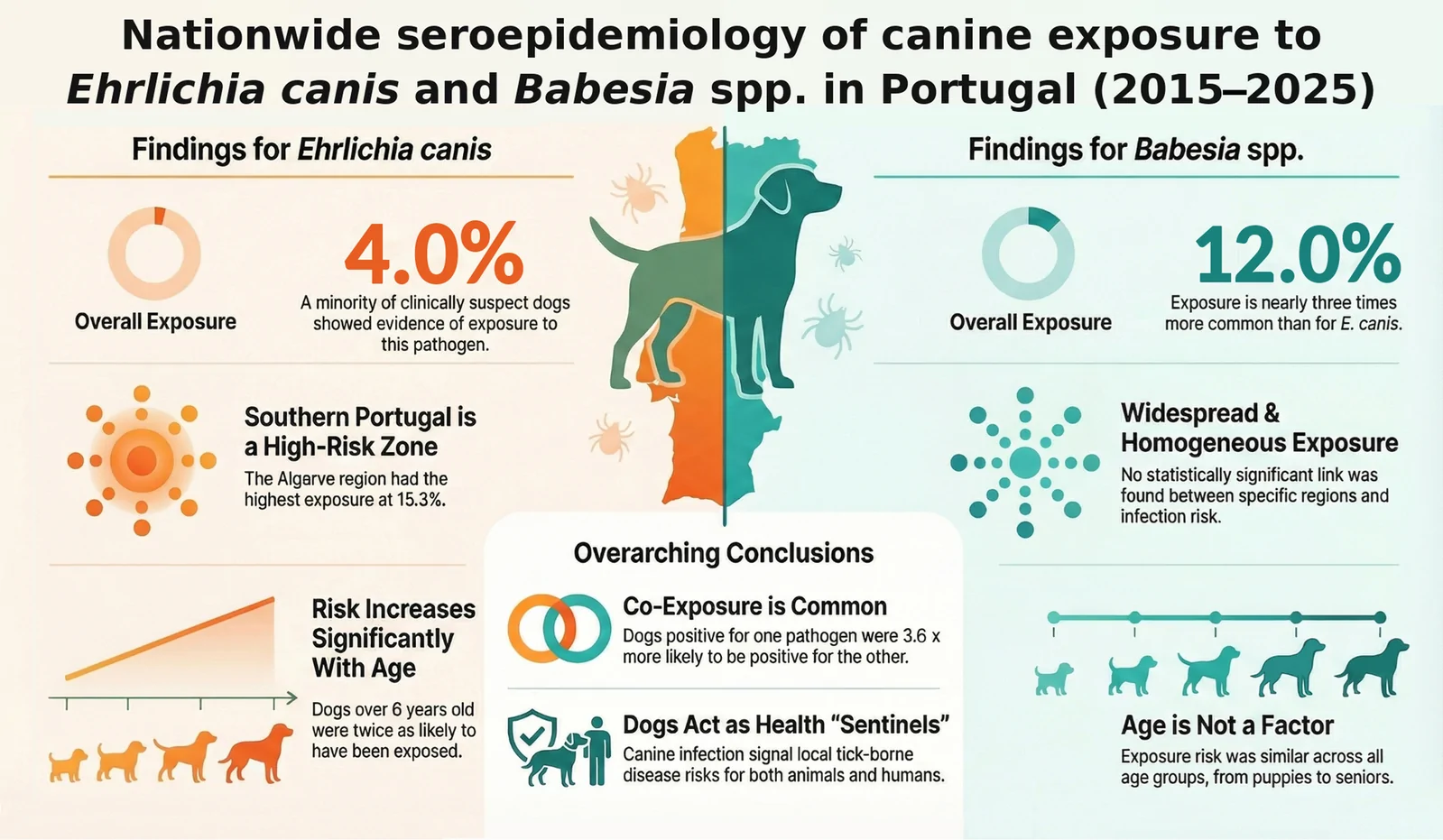

## Background

Canine vector-borne diseases (CVBD) comprise a globally distributed group of infections caused by a wide spectrum of viruses, bacteria, protozoa and helminths transmitted by hematophagous arthropods. These diseases represent a major cause of canine morbidity and mortality, with important zoonotic implications [1–11]. Many CVBD agents circulate in endemic foci across the Mediterranean basin and the Iberian Peninsula. In these settings, climatic conditions suitable for competent vectors, together with close interfaces between dogs, wildlife and humans, facilitate pathogen circulation and exposure [1–4, 9, 10, 12–16]. In this context, ixodid ticks are key vectors for multiple CVBD pathogens. Portugal harbours at least 20 hard-tick species across five genera (*Dermacentor*, *Haemaphysalis*, *Hyalomma*, *Ixodes*, and *Rhipicephalus*). Their distribution and ecology appear increasingly shaped by climate trends and anthropogenic landscape change [2, 14, 17–19]. Among these, the brown dog tick, *Rhipicephalus sanguineus* sensu lato (s.l.), is consistently reported as the predominant tick infesting dogs and constitutes a central vector for agents infecting both animals and humans in Portugal [2, 4, 7–9, 11, 13, 14, 18, 20–24].

Within the tick-borne agents of major veterinary and zoonotic relevance in Portugal, *Ehrlichia canis* (the aetiological agent of canine monocytic ehrlichiosis, CME) and apicomplexan piroplasmids of the genus *Babesia* represent cornerstone pathogens [2, 4, 6, 8, 11, 14, 17, 20, 24, 25]. *Ehrlichia canis* is primarily transmitted by *R. sanguineus* s.l. [2, 7, 8, 20, 24, 26], and has been molecularly confirmed in Portuguese dogs [1–3, 7, 11, 14, 24, 27]. Early serological surveys conducted in different southern regions reported high seroprevalence of *E. canis* among clinically suspected dogs, indicating previous exposure rather than necessarily active infection, including 23.8% [7] and 24.8% [10] in the Algarve, and 25.4% in the Alentejo regions [7]. Most recent national data support a similar geographical gradient, with significantly higher prevalence in southern NUTS level 2 regions, such as the Algarve (13.9%) and Alentejo [8, 11]. Nevertheless, a molecular investigation targeting the temperate lineage of *R. sanguineus* s.l., the one predominantly detected in Portugal, reported no *E. canis* DNA in screened ticks, raising questions regarding vector competence and/or the contribution of additional vectors or lineages under field conditions [28, 29].

Piroplasmids infecting erythrocytes have been traditionally categorised morphologically into large (~ 3 − 5 µm) or small (~ 0.5 − 2.5 µm) piroplasms. However, molecular tools are essential for accurate species-level identification, given marked differences in virulence, vector specificity, geographical distribution and drug susceptibility [17]. It is also important to recognise that serology primarily reflects exposure rather than active infection and does not provide species identification [15–17]. Molecular studies in Portugal have documented the circulation of large piroplasms, including *Babesia canis* (vector: *Dermacentor reticulatus*) and *Babesia vogeli* (vector: *R. sanguineus* s.l.) in dogs [2, 30]. The first molecular identification of *B. canis* and *B. vogeli* in Portugal was reported from the northern region [30]. The small piroplasm *Babesia vulpes* has also been confirmed in Portuguese dogs, with early cases suggesting either vertical transmission or autochthonous tick-borne infection [5, 31]. Reported *Babesia* spp. seroprevalence in Portugal has ranged from 3% in military working dogs [2] to 58% in kennel/shelter dogs in the Algarve [10], a circumstance supporting endemicity. Tick-based surveys further corroborate the circulation of these and other protozoal agents, reporting molecular detection of *Babesia* spp. in collected ticks [13, 14].

In addition to *E. canis* and *Babesia* spp., a broad array of zoonotic and veterinary pathogens has been documented in Portuguese dogs and ticks. Notably *Rickettsia conorii* (agent of Mediterranean spotted fever [MSF]), transmitted by *R. sanguineus* s.l., has reached seroprevalence levels of up to 69% in kennel/shelter dog surveys [23] and, more recently, 27.0% overall in a nationwide serological study, peaking at 48.0% in the Algarve [16]. Other agents detected in Portuguese dogs and/or ticks include *Anaplasma* spp. (notably *A. platys* and *A. phagocytophilum*), *Hepatozoon* spp., *Borrelia* spp. (including *B. burgdorferi* s.l. and relapsing fever-like *Borrelia*), *Cercopithifilaria* spp., and several spotted-fever group *Rickettsia* spp. (e.g., *R. aeschlimannii*, *R. helvetica*, *R. massiliae*, *R. raoultii* and *R. slovaca*), alongside the molecular detection of *Theileria annulata* and *Theileria ovis* in hard ticks collected from domestic animals and wildlife in Portugal [1, 13, 14].

Co-exposure and co-infection with multiple CVBD agents, including *E. canis* and *Babesia* spp., are frequently reported in endemic areas [7], complicating clinical interpretation and potentially exacerbating disease severity [10]. Nationwide serological data indicate that a substantial proportion of Portuguese dogs (up to 46.3% of clinically suspected dogs) show evidence of exposure to at least one vector-borne agent [2, 7]. However, long-term, nationwide seroepidemiologic studies concurrently evaluating host and environment-related determinants of exposure to both *E. canis* and *Babesia* spp. remain limited [2]. Ecological niche modelling further supports the plausibility of large-scale surveillance, implicating *R. sanguineus* s.l. as a major contributor to predicted risk for ehrlichiosis and anaplasmosis across the Iberian Peninsula [8]. Given that vector distribution and activity (notably *R. sanguineus* s.l. and *D. reticulatus*) are strongly modulated by regional climatic and ecological conditions in Portugal, integrated assessment of exposure determinants is crucial to inform targeted prevention and One Health-oriented surveillance [1–5, 10, 13, 14, 16–18, 20, 23, 30, 32].

Accordingly, this study presents a nationwide, laboratory-based retrospective assessment of seropositivity to *E. canis* and *Babesia* spp. in dogs submitted for diagnostic testing under clinical suspicion of tick-borne disease in mainland Portugal and the autonomous regions over an 11-year period (2015–2025). The objectives were to describe patterns of serological exposure in this clinically selected population and to explore host- and geography-related factors associated with seropositivity, thereby generating evidence to support risk-based tick control and surveillance strategies within a One Health perspective. We hypothesised that seropositivity to *E. canis* and *Babesia* spp. would vary according to host- and geography-related characteristics in clinically suspect dogs from Portugal.

## Methods

### Sampling, data collection and diagnostic procedures

This was a retrospective, laboratory-based observational study based on convenience serum samples from dogs submitted to CEDIVET Veterinary Laboratories (Portugal) for diagnostic testing between 2015 and 2025. Samples originated from dogs in which a tick-borne disease was clinically suspected by the attending veterinarians at the submitting veterinary centres. Because this was a retrospective laboratory-based dataset, no standardised case definition for clinical suspicion and no detailed clinical metadata were available. Their anonymous use for research purposes was authorised under Institutional Review Board approval from CEDIVET Veterinary Laboratories (protocol code CEDIVET.005/2023), covering samples submitted by participating veterinary clinics and hospitals. A total of 2960 samples were obtained from 234 veterinary medical centres, including clinics and hospitals, across mainland Portugal and the insular autonomous regions. All eligible residual serum samples meeting the inclusion criteria during the study period were included, and no random subsampling was performed. No a priori sample size calculation was performed given the retrospective nature of the study, based on all eligible archived residual diagnostic samples available during the study period. Each submission was accompanied by a laboratory request form containing basic signalment details, namely, breed, sex, and age, with all remaining data anonymised. Only samples with the complete set of variables were included. Still, dogs lacking age information (17.2% of the dataset) were retained for overall analyses but excluded from age-stratified analyses. Mixed-breed dogs were omitted from breed and size-specific assessments. Breeds were categorised into five size groups: (a) toy (< 5 kg), (b) small (6–10 kg), (c) medium (11–25 kg), (d) large (26–40 kg) and (e) giant (41 kg or more) [16, 33]. For analytical purposes, age was categorised into five groups: (a) puppy, < 1 year; (b) young, 1 to < 2 years; (c) adult, 2 to < 6 years; (d) senior, 6 to < 11 years; and (e) geriatric, ≥ 11 years [15, 16, 34]. After routine diagnostic processing, serum samples were refrigerated at 2–8 °C until analysis and subsequently stored at −20 °C for archival purposes when residual material was available.

Serum samples were analysed for the presence of *E. canis* and *Babesia* spp. antibodies as follows:

To detect immunoglobulin G (IgG) antibodies to *E. canis*, canine sera were diluted at 1:100 in sample buffer and examined for the qualitative detection of circulating IgG antibodies for *E. canis* with the Euroimmun^®^ test (Euroimmun Medizinische Labordiagnostika AG, Lübeck, Germany). The reported sensitivity and specificity of the Euroimmun^®^ test were 92% and 100%, respectively. An enzyme-linked immunosorbent assay (ELISA) was performed in accordance with the manufacturer’s instructions. Optical density ratios were classified into three ascending categories: samples with a ratio < 0.8 were interpreted as negative, those between ≥ 0.8 and < 1.1 were considered borderline, and those with a ratio ≥ 1.1 were considered positive [11].

To detect IgG antibodies against *Babesia* spp., a commercial ELISA kit (*Babesia* ELISA Dog, Afosa, Blankenfelde-Mahlow, Germany) was used. The manufacturer’s reference ranges were defined as follows: negative (< 14), borderline (14 − 19), and positive (> 19). According to the manufacturer, the diagnostic sensitivity and specificity of this assay for *B. canis,* when compared with the indirect immunofluorescence assay, are 91.6% and 95.4%, respectively. Nevertheless, cross-reactivity with other *Babesia* spp. (i.e., *B. vogel*i and *B. gibsoni*) as well as with the related piroplasm *Rangelia vitalii* has been reported [35]; therefore, the detected antibodies are herein referred collectively as antibodies to *Babesia* spp. [36].

Borderline ELISA results were considered indeterminate according to the manufacturers’ thresholds and were excluded from inferential analyses to avoid forced misclassification. Analyses were restricted to definitive (positive/negative) outcomes.

### Statistical analysis

All statistical analyses were performed using JMP^®^ Pro version 18.0.2 (SAS Institute Inc., Cary, NC, USA), MedCalc^®^ version 20.006 (MedCalc Software Ltd, Ostend, Belgium) and DATAtab^®^ (numiqo e.U. Graz, Austria). The dataset exported from the Sislab^®^ laboratory management system (Glintt, Global Intelligent Technologies, Lisbon, Portugal) was verified for consistency, completeness and integrity prior to analysis.

Categorical variables included geographical region (NUTS level 2), district, season, month, breed, breed size, sex and age group, while continuous antibody optical density ratios were classified according to manufacturer-defined thresholds. The primary outcomes were definitive ELISA seropositivity (positive versus negative) for *E. canis* and *Babesia* spp. Descriptive statistics were generated for all variables, and seropositivity was expressed as a proportion with exact binomial 95% confidence intervals (CI).

Associations between seropositivity and categorical predictors were examined using the Pearson chi-square test (*χ*^2^), with Yates’ correction where appropriate and Cramér’s V to assess effect size. Fisher’s exact test was applied when expected frequencies were < 5. Statistical significance was defined as *P* < 0.05. Given the exploratory nature of the study and the number of univariable comparisons performed, unadjusted *p* values were interpreted cautiously and in conjunction with effect sizes, 95% CI and biological plausibility. No formal multiplicity adjustment was applied.

Potential risk factors were explored using univariable logistic regression, estimating odds ratios (OR) and 95% CI for each explanatory variable. Reference categories were defined as follows: puppies (< 1 year) for age, females for sex, toy-sized breeds (< 5 kg) for size, Porto for district, the North for NUTS 2 region, and winter for season. Variables with *P* ≤ 0.20 in univariable screening were subsequently entered into a multivariable logistic regression model, retaining predictors with *P* < 0.05. Model refinement was assessed with the likelihood-ratio test, and model adequacy with the Hosmer–Lemeshow goodness-of-fit, Nagelkerke’s R^2^, and the area under the receiver operating characteristic curve (AUC).

Model residuals were examined to identify potential outliers or influential data points. All assumptions of logistic regression were verified. Results were reported as adjusted odds ratios (aOR) with 95% CI and corresponding *p* values. Statistical significance was established at the conventional α level of 0.05 [16, 37].

## Results

### General seropositivity, geographical distribution and seasons

Of the 2960 canine serum samples analysed, 116 (3.9%) samples were seropositive, 2756 (93.1%) seronegative, and 88 (3.0%) borderline for *E. canis* IgG antibodies (ELISA). For *Babesia* spp., 337 (11.4%) samples tested seropositive, 2481 (83.8%) seronegative, and 142 (4.8%) borderline for IgG antibodies (ELISA). The serological results for both agents are summarised in Table 1. Co-seropositivity was identified in 32 samples (1.1%), while 2345 samples tested negative for both pathogens, indicating that concurrent or cross-reactive serological responses were uncommon and accounted for fewer than 2% of all cases. To enhance interpretability, further analyses were restricted to definitive ELISA outcomes (positive/negative), and borderline results were excluded from denominator calculations and subsequent modelling.

**Table 1:** Cross-tabulation of *Ehrlichia canis* and *Babesia* spp. and ELISA results in 2,960 canine serum samples

		*Ehrlichia* canis Ac (ELISA)			
		Negative * n* (%)	Borderline * n* (%)	Positive * n* (%)	Total* n* (%)
*Babesia* spp. Ac (ELISA)	Negative	2345 (79.2)	65 (2.2)	71 (2.4)	2481 (83.8)
	Borderline	123 (4.2)	6 (0.2)	13 (0.4)	142 (4.8)
	Positive	288 (9.7)	17 (0.6)	32 (1.1)	337 (11.4)
	Total	2756 (93.1)	88 (3.0)	116 (3.9)	2960 (100)

*Ac*, antibodies; *ELISA*, enzyme-linked immunosorbent assay

Most samples originated from the districts of Porto (*n* = 1430; 48.3%), Viseu (*n* = 532; 18.0%), Aveiro (*n* = 245; 8.3%), and Coimbra (*n* = 236; 8.0%). The spatial distribution of *E. canis* and *Babesia* spp. seropositivity across the NUTS 2 regions of mainland Portugal and the autonomous regions is presented in Table 2. Both agents exhibited marked geographical variation.

**Table 2:** Seropositivity to *Ehrlichia canis* and *Babesia* spp. ELISA results by region (NUTS 2)

	*Ehrlichia* canis Ac (ELISA)			*Babesia* spp. Ac (ELISA)		
Region (NUTS 2)	*n* of dogs tested (%)	% seropositive dogs^a^ (*n*)	95% CI	*n* of dogs tested (%)	% seropositive dogs^b^ (*n*)	95% CI
North	1667 (58.0)	4.0 (67)	3.2–5.1	1626 (57.7)	12.2 (199)	10.7–13.9
Centre	1030 (35.9)	2.7 (28)	1.9–3.9	1026 (36.4)	11.7 (120)	9.9–13.8
Algarve	98 (3.4)	15.3 (15)	9.5–23.7	92 (3.3)	12.0 (11)	6.8–20.2
Autonomous Region of Madeira	26 (0.9)	7.7 (2)	2.1–24.1	25 (0.9)	4.0 (1)	0.7–19.5
Alentejo	28 (1.0)	7.1 (2)	2.0–22.6	27 (1.0)	18.5 (5)	8.2–36.7
Greater Lisbon	11 (0.4)	9.1 (1)	1.6–37.7	10 (0.4)	0.0 (0)	0.0–27.8
Autonomous Region of the Azores	9 (0.3)	0.0 (0)	0.0–29.9	9 (0.3)	11.1 (1)	2.0–43.5
Setúbal Peninsula	2 (0.1)	50.0 (1)	9.5–90.5	2 (0.1)	0.0 (0)	0.0–65.8
West and Tagus Valley	1 (0.0)	0.0 (0)	0.0–79.3	1 (0.0)	0.0 (0)	0.0–79.3
Total	2872 (100.0)	4.0^c^ (116)	3.4–4.8	2818 (100.0)	12.0^c^ (337)	10.8–13.2

*Ac*, antibodies; *CI*, confidence interval; *ELISA*, enzyme-linked immunosorbent assay; *NUTS 2*, Nomenclature of Territorial Units for Statistics level 2

^a^ *χ2* = 50.37, *df* = 8, *P* =  < 0.001*

^b^ *χ2* = 4.57, *df* = 8, *P* = 0.803

^c^*χ2* = 38.89, *df* = 1, *P* < 0.001*

^*^Statistically significant

**Fig. 1:**
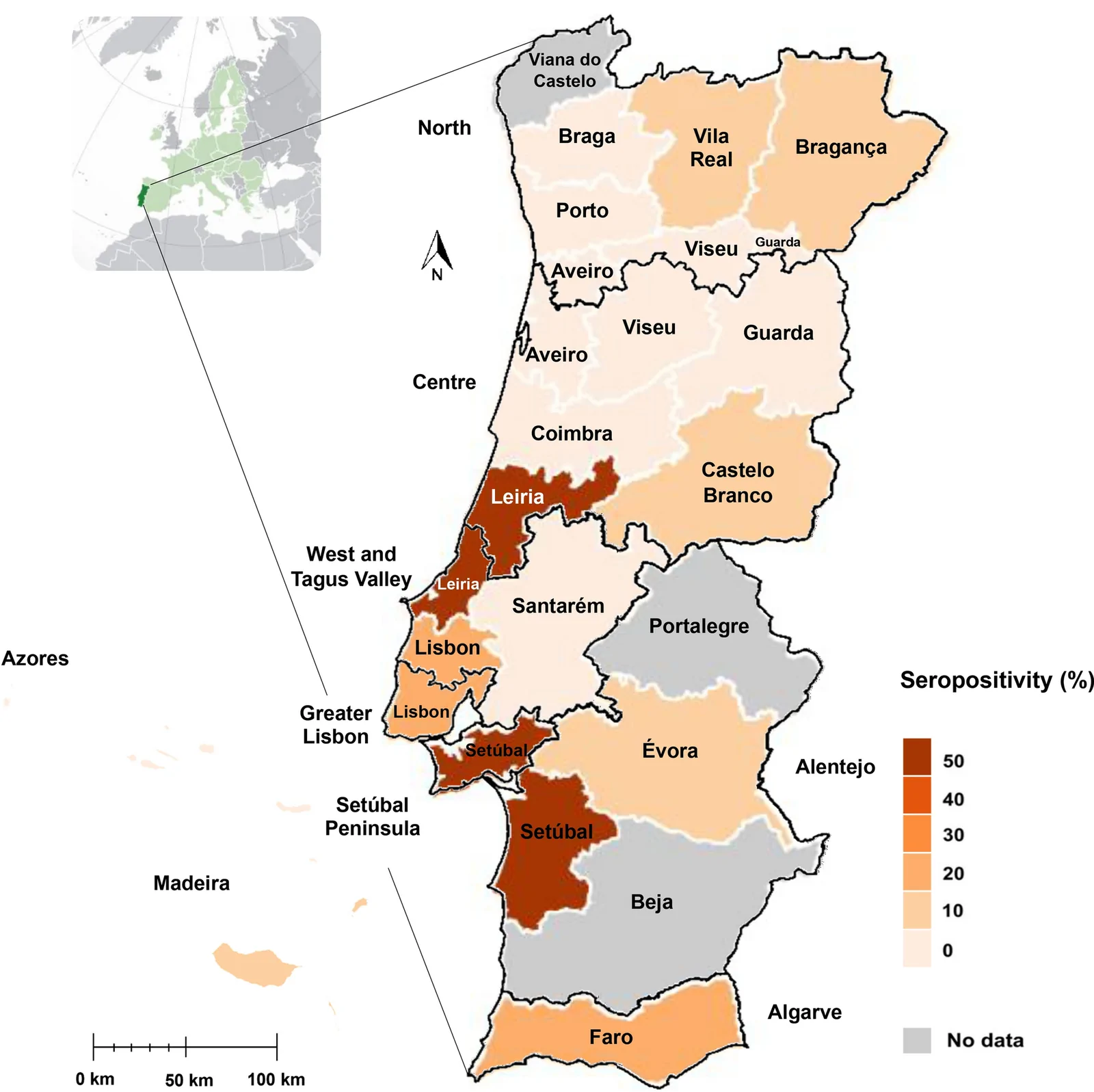
Map of Portugal showing categorical representation of *Ehrlichia canis* seropositivity over 11 years (2015–2025), determined by ELISA per district and NUTS 2 (map drawn in: www.mapinseconds.com; accessed on 15 December 2025). Seropositivity values in districts with very low sample sizes (*n* ≤ 5) are not statistically robust and should be interpreted with caution. Uneven sample distribution may also reflect regional differences in clinical suspicion or awareness of *E. canis*. ELISA, enzyme-linked immunosorbent assay, NUTS 2, Nomenclature of Territorial Units for Statistics level 2

Seropositivity to *E. canis* ranged from 0.0% to 15.3%, with an overall seropositivity of 4.0% based on definitive ELISA results. The highest proportions of positive samples were detected in the Algarve (15.3%), followed by the Greater Lisbon region (9.1%), the Autonomous Region of Madeira (7.7%) and the Alentejo (7.1%). Intermediate values were observed in the North (4.0%) and Centre (2.7%) regions, while no positive samples were recorded in the Autonomous Region of the Azores or in the West and Tagus Valley regions. The chi-square test revealed a statistically significant association between NUTS 2 regions and *E. canis* seropositivity (*χ*^2^ = 50.37, *df* = 8, *P* < 0.001; Cramér’s V = 0.19). Spatial heterogeneity in seropositivity across Portuguese districts and NUTS 2 regions is summarised in Fig. 1.

The Algarve exhibited the highest odds of *E. canis* seropositivity (OR = 4.3, 95% CI 2.4–7.9, *P* < 0.001), followed by the Setúbal Peninsula (OR = 23.9, 95% CI 1.5–385.9, *P* = 0.025). The North region served as the reference category (4.0%; OR = 1). The elevated OR observed in the Setúbal Peninsula should be interpreted with caution due to the very limited sample size (*n* = 2). This estimate should, therefore, be regarded as descriptive and not interpreted as robust evidence of increased regional odds (Table 2).

Seropositivity to *Babesia* spp. also varied geographically, ranging from 0.0% to 18.5%, with an overall seropositivity of 12.0% based on definitive ELISA results. The highest proportions of positive samples were observed in the Alentejo (18.5%), followed by the North (12.2%), Centre (11.7%), Algarve (12.0%) and the Autonomous Region of the Azores (11.1%). Lower values were recorded in the Autonomous Region of Madeira (4.0%), and no positive samples were identified in Greater Lisbon, the Setúbal Peninsula, or the West and Tagus Valley regions. The chi-square test revealed no statistically significant association between NUTS 2 regions and *Babesia* spp. seropositivity (*χ*^2^ = 4.57, *df* = 8, *P* = 0.803; Cramér’s V = 0.06), indicating that no statistically significant regional association was detected in this dataset. This finding should be interpreted cautiously given the uneven regional sampling and sparse strata in several regions (Table 2).

Considering the definitive ELISA results, a statistically significant difference was found between the seropositivity of 4.0% to *E. canis* and that of 12.0% to *Babesia* spp. (*χ*^2^ = 38.89, *df* = 1, *P* < 0.001; Cramér’s V = 0.12). Furthermore, seropositivity to *Babesia* spp. was a risk factor for seropositivity to *E. canis* (OR = 3.6, CI 2.3–5.5, *P* < 0.001).

No statistically significant associations were detected between *E. canis* seropositivity and either season (*χ*^2^ = 2.06, *df* = 3, *P* = 0.561; Cramér’s V = 0.04) or month (*χ*^2^ = 9.22, *df* = 11, *P* = 0.602; Cramér’s V = 0.08). Similarly, *Babesia* spp. seropositivity showed no significant association with season (*χ*^2^ = 4.97, *df* = 3, *P* = 0.174; Cramér’s V = 0.06) or month (*χ*^2^ = 8.36, *df* = 11, *P* = 0.680; Cramér’s V = 0.08).

These findings indicate that, within the study period, *E. canis* and *Babesia* spp. exposures were relatively homogeneous throughout the year, showing no clear evidence of seasonal clustering or month-specific variation in infection odds. However, this should be interpreted cautiously given the clinically selected nature of the dataset and the limitations inherent to retrospective laboratory-based series.

### Dog size

For the analysis of dog size, mixed-breed dogs were excluded, resulting in 1195 dogs for *E. canis* and 1165 for *Babesia* spp. distributed across five size categories (toy, small, medium, large, and giant).

For *E. canis*, seropositivity ranged from 8.3% in toy breeds to 3.9% in giant breeds, with intermediate values in small (3.9%), medium (5.2%), and large (4.3%) dogs. The chi-square test revealed no statistically significant association between breed size and *E. canis* seropositivity (*χ*^2^ = 4.23, *df* = 4, *P* = 0.375; Cramér’s V = 0.08), indicating that no statistically significant association with size category was detected in this dataset.

Similarly, *Babesia* spp. seropositivity ranged from 9.6% in toy breeds to 13.0% in giant breeds, with the highest proportion observed in large dogs (*n* = 61; 11.5%). The chi-square test also showed no significant association between breed size and *Babesia* spp. seropositivity (*χ*^2^ = 0.96, *df* = 4, *P* = 0.915; Cramér’s V = 0.04), suggesting that no size-associated difference in *Babesia* spp. seropositivity was detected.

Overall, no evidence was found of size-dependent variation in exposure to either pathogen.

### Breed

The study population comprised 2,960 dogs representing 89 recognised breeds. Mixed-breed dogs were predominant (*n* = 1704; 57.6%), followed by Labrador Retriever (*n* = 207; 7.0%) and German Shepherd (*n* = 127; 4.3%). For breed-specific analyses, mixed-breed dogs were excluded to reduce heterogeneity, resulting in 1,256 purebred dogs being included in the comparisons. No statistically significant association was identified between breed and seropositivity to either *E. canis* (*χ*^2^ = 75.6, *df* = 87, *P* = 0.804; Cramér’s V = 0.34) or *Babesia* spp. (*χ*^2^ = 97.3, *df* = 87, *P* = 0.212; Cramér’s V = 0.39).

Collectively, these findings did not provide evidence of a statistically significant breed-associated difference to either *E. canis* or *Babesia* spp. in Portuguese dogs.

### Sex

No statistically significant differences were observed between females and males for either *E. canis* or *Babesia* spp.

For *E. canis*, males showed slightly higher seropositivity (4.5%) than females (3.5%), but this difference was not significant (*χ*^2^ = 1.71, *df* = 1, *P* = 0.191; Cramér’s V = 0.02). Similarly, *Babesia* spp. seropositivity was comparable between males (12.4%) and females (11.4%), with no significant association (*χ*^2^ = 0.66, *df* = 1, *P* = 0.416; Cramér’s V = 0.02).

### Age

Age data were available for 2451 of the 2960 dogs analysed. A total of 509 dogs (17.2%) lacked age information in the requisition form and were, therefore, excluded from age-stratified analyses. Age distribution among these 2451 dogs ranged from 6 months (≤ 1 year) to 20 years (≥ 11 years), with a median age of 5 years (interquartile range: 2.0–8.1 years).

For *E. canis*, the chi-square test revealed a statistically significant association between age group and seropositivity (*χ*^2^ = 12.5, *df* = 4, *P* = 0.014; Cramér’s V = 0.10). Puppies (< 1 year) were used as the reference category (10.4%; OR = 1.0). Logistic regression analysis showed that dogs aged 6 to < 11 years (26.8%; OR = 2.1, 95% CI 1.3 − 3.5, *P* = 0.004) and those aged ≥ 11 years (9.7%; OR = 2.1, 95% CI 1.1 − 4.1, *P* = 0.020) had significantly higher odds of seropositivity compared with puppies, a circumstance consistent with cumulative exposure over time. No significant differences were observed among younger age groups (1 to < 2 years, 9.5%; OR = 0.6; 2 to < 6 years, 26.4%; OR = 1.1).

In contrast, no statistically significant association was found between age group and seropositivity to *Babesia* spp. (*χ*^2^ = 2.38, *df* = 4, *P* = 0.667; Cramér’s V = 0.05). Seropositivity was relatively homogeneous across age classes, ranging from 11.0% to 13.0%, indicating that no age-associated difference in *Babesia* spp. seropositivity was detected in this dataset.

### Multivariable logistic regression analysis

Multivariable logistic regression models were constructed to identify factors associated with seropositivity to *E. canis* and *Babesia* spp. The dependent variables were definitive ELISA outcomes for each pathogen. Given the limited number of positive events, sparse regional strata and incomplete availability of management-related covariates, these models were interpreted as exploratory rather than confirmatory.

Both multivariable models showed moderate discrimination (*χ*^2^ = 96.2, *df* = 90, *P* = 0.308; Nagelkerke R^2^ = 0.15; AUC = 0.71). There was no evidence of gross lack of fit on goodness-of-fit testing (*P* > 0.05), although calibration and effect estimates were limited by sparse strata.

In the *E. canis* model, no predictor reached statistical significance. Dogs from the Setúbal district had higher odds of seropositivity than those from Porto, but this association was not statistically significant and should be interpreted cautiously because of sparse data (Table 2).

In the *Babesia* spp. model, no predictor was statistically significant, and the overall distribution of effects was comparable to that observed for *E. canis*.

Taken together, these findings suggest that patterns of serological exposure to *E. canis* and *Babesia* spp. in Portugal may be influenced by vector-related and ecological factors. However, no predictor remained independently associated with seropositivity after multivariable adjustment, suggesting limited explanatory capacity of the available variables rather than absence of biologically relevant determinants of exposure.

## Discussion

### General seropositivity and geographical distribution

In this nationwide, laboratory-based retrospective study of dogs tested because of clinical suspicion of tick-borne disease (2015–2025; *n* = 2960), serological evidence of exposure to *Babesia* spp. was more frequent than exposure to *E. canis* (Table 2). When analyses were restricted to definitive ELISA outcomes, the overall seropositivity was 12.0% for *Babesia* spp. and 4.0% for *E. canis*, with co-seropositivity detected in only 1.1% of dogs. These findings should not be interpreted as estimates of population prevalence, as the study was based on a clinically selected convenience sample of dogs submitted for diagnostic testing. Rather, they reflect serological patterns of exposure within a diagnostic population. Within this context, exposure to canine piroplasms appeared more frequent than exposure to *E. canis* in Portugal, although comparisons with earlier Portuguese reports should be interpreted cautiously given heterogeneity in study design, diagnostic platforms and target populations [1–4, 7].

The seropositivity of *E. canis* in this Portuguese cohort is lower than that reported in several Mediterranean and subtropical regions, where values frequently exceed 10–20% and may reach as high as 82% in hyperendemic foci, such as Cape Verde [22], or 27% in Namibia [38]. In contrast, studies from Central and Northern Europe, as well as some urbanised Mediterranean settings, often report lower values, typically below 10% [21, 39–41].

Regarding *Babesia* spp., the observed seropositivity of 12.0% is broadly concordant with global pooled estimates (~ 12%) and compatible with the burden expected in endemic European contexts [42]. Nevertheless, direct comparisons across studies should be interpreted with caution: in Southern Europe and the Mediterranean basin, reported seroprevalence typically spans a wide range (~ 1–16%), reflecting genuine geographical variation in ecological and vector-related risk, as well as methodological and sampling differences (diagnostic platform and cutoff; clinically suspect versus general population; owned dogs versus shelter/stray cohorts) that collectively drive substantial between-study heterogeneity [21, 41, 43]. In line with this, a recent global systematic review and meta-analysis reported Europe as the region with the highest pooled prevalence (20.7%; 95% CI 9.7–34.4) and identified *B. canis* as the most prevalent species (21.6%; 95% CI 5.6–44.1) [42].

In the present study, a key finding was the marked geographical heterogeneity for *E. canis*, with significantly higher seropositivity in southern regions, particularly the Algarve (15.3%) and Alentejo (7.1%), as well as Greater Lisbon (9.1%) and the Autonomous Region of Madeira (7.7%), while no positive samples were recorded in the Azores or West and Tagus Valley. This “southward gradient” is biologically plausible given the recognised ecology of *R. sanguineus* s.l., a primary vector for *E. canis*, which is favoured by warmer and drier conditions and may exhibit prolonged seasonal activity in Mediterranean climates [8, 18, 26]. Entomological updates and pathogen surveys in Portugal further corroborate that competent tick species and multiple tick-borne agents occur across the country, but with regionally variable distributions and pathogen associations [4, 13, 14, 18, 23].

The extremely high estimate for the Setúbal Peninsula (50%) should be interpreted cautiously, because it is driven by a very small subsample (*n* = 2), resulting in a wide CI and an inflated OR, an expected limitation in sparse-data epidemiology [37]. Nonetheless, the broader pattern (higher *E. canis* exposure in warmer southern areas) is consistent with Iberian risk modelling for ehrlichiosis/anaplasmosis and the notion that macroclimate and microhabitat suitability shape *R. sanguineus* s.l. abundance and transmission intensity [8, 18].

In contrast, *Babesia* spp. seropositivity showed no statistically significant association with NUTS 2 region, despite numerical variability (0.0–18.5%). Several non-mutually exclusive explanations merit consideration. First, *Babesia* spp. in this study represents a serological grouping that may include exposure to multiple canine piroplasms (including *B. canis*, *B. vogeli* and potentially others), and pooling can dilute region-specific signals for individual species [17, 20, 30, 43]. Second, *Babesia* transmission may involve broader or partially distinct ecological pathways compared with *E. canis*, including different vectors in some contexts, which may reduce the ability to detect region-specific signals at the NUTS 2 level [17, 43]. Third, absence of positives in specific regions (e.g., Greater Lisbon) in this dataset may reflect local sampling structure and referral/testing behaviours rather than true absence of exposure, particularly when strata sizes are modest [37, 44].

No clear seasonal or month-specific clustering was identified for either pathogen in our dataset. This should not be taken to exclude seasonal transmission, but rather to reflect the intrinsic limitations of long-term, clinically driven laboratory series in resolving temporal peaks. In a recent global systematic review and meta-analysis, canine *Babesia* spp. prevalence was highest in summer (9.7%; 95% CI 4.0–17.4) while also documenting substantial between-study heterogeneity [42]. In Europe, seasonal dynamics may be further shaped by vector ecology and local climate, interannual variability, changing preventive practices, and peridomestic tick activity may collectively smooth or obscure seasonal patterns in submitted samples [4, 18, 42, 43].

Finally, seropositivity to *Babesia* spp. was a risk factor for seropositivity to *E. canis* (OR = 3.6, CI 2.3–5.5), an epidemiologically plausible pattern consistent with shared tick-borne exposure pathways and with evidence that dogs and their ticks in endemic settings frequently harbour multiple pathogens [14, 23, 45, 46]. Co-infection is clinically important, because it can amplify case severity, alter hematological profiles, and complicate interpretation of diagnostic results and therapeutic response [29, 45, 47–50].

### Dog size-associated patterns

No significant association was detected between dog size categories and seropositivity for either pathogen. This supports the concept that, at a population level, body size is not a primary biological determinant of exposure to tick-borne agents; rather, exposure is usually mediated by extrinsic factors, such as environment (urban/periurban/rural), roaming behaviour, and use/adherence to tick preventatives [7, 21, 44]. The absence of a size effect in a clinically suspect cohort also argues against a simple “large dog, more ticks” heuristic interpretation and instead points back to vector ecology and management as dominant drivers [8, 18].

### Breed-associated patterns

Breed was not significantly associated with seropositivity to either *E. canis* or *Babesia* spp. This finding is consistent with much of the vector-borne disease literature, in which apparent breed risks often reflect confounding by management practices (e.g., hunting, outdoor housing), geography, and preventive regimes rather than intrinsic genetic susceptibility [7, 21, 44]. In practice, breed-level differences may emerge in narrowly defined subpopulations (e.g., working dogs, shelter populations) because of differential tick exposure and preventive access rather than intrinsic breed susceptibility [2, 11].

### Sex-associated patterns

Sex was not a significant predictor of seropositivity, although males showed a non-significant tendency towards higher *E. canis* seropositivity. The broader literature is inconsistent regarding sex effects, and where differences exist, they are frequently attributed to behavioural/management confounding (e.g., roaming, outdoor activity, neuter status) rather than sex-linked immunobiology [38, 44, 49]. The absence of a clear sex signal here is, therefore, unsurprising and is consistent with the likely importance of vector, environmental and management-related factors [4, 21].

### Age-associated patterns

Age showed a significant relationship with *E. canis* seropositivity, with older dogs (≥ 6 years) demonstrating increased odds compared with puppies. This is most plausibly explained by cumulative tick exposure over time and the persistence of antibodies to *Ehrlichia* following infection, which can cause serology to reflect historical exposure rather than active infection [32, 38, 44]. Similar age-associated increases in exposure have been documented across endemic settings, supporting the interpretation that repeated infestations and reinfections accumulate with time-at-risk [38, 49].

By contrast, *Babesia* spp. seropositivity did not vary significantly by age. In endemic areas, early-life exposure can occur rapidly and may homogenise seropositivity across age strata, particularly when dogs encounter ticks soon after weaning or adoption [21, 43]. In addition, because the assay detects IgG to *Babesia* spp. collectively, heterogeneity by age for individual species could be masked [17, 30].

### One Health perspectives and public health relevance

Although *E. canis* and canine *Babesia* spp. are primarily veterinary pathogens, their epidemiology is tightly linked to human health through shared vectors, peridomestic transmission settings, and overlapping exposure pathways. Dogs may, therefore, serve as practical biosentinels of local tick-borne exposure, although the present study did not include parallel human, tick or environmental sampling. In northern Portugal, a 2024 shelter-dog serosurvey documented substantial exposure to *E. canis* alongside *R. conorii*, reinforcing that canine seropositivity can signal heightened human exposure risk in the same environments [11]. Importantly, uncommon human infection with *E. canis* has been documented, as identical *E. canis* sequences in an infected patient and an attached tick were confirmed in Italy [51], underlining the One Health value of integrated tick control and surveillance at the animal–human interface. By contrast, the *Babesia* spp. most frequently infecting dogs are not the principal agents of human babesiosis in Europe, which is typically attributed to *Babesia divergens*, *Babesia microti* and *Babesia venatorum*. Importantly, there is currently no published evidence demonstrating cross-reactivity of the canine ELISA used in this study with these zoonotic *Babesia* spp. in canine sera. Accordingly, seropositivity in dogs should be interpreted cautiously as evidence of exposure to *Babesia* spp. rather than to any specific zoonotic species [52].

## Limitations of this study

Several limitations should be explicitly acknowledged when interpreting these findings.

1. Serology measures exposure, not current infection. IgG positivity cannot reliably distinguish active infection from past exposure, particularly when antibodies may persist after clinical recovery or subclinical infection [32, 44]. This limitation is especially relevant for retrospective laboratory datasets lacking contemporaneous clinical staging and treatment history [44].
2. Potential cross-reactivity and agent pooling: The *Babesia* ELISA employed is known to exhibit cross-reactivity with other piroplasms in some settings. Therefore, reporting as “anti-*Babesia* spp.” is appropriate but reduces species-level epidemiological resolution [17, 36, 43]. Species-level inference is particularly important in Portugal, where multiple canine piroplasms have been documented, including *B. canis* and *B. vogeli* [5, 20, 30].
3. Handling of borderline ELISA results: Borderline results were excluded from the primary inferential analyses, because they were considered indeterminate according to the manufacturers’ thresholds. This approach reduced forced misclassification but may have influenced seropositivity estimates and statistical power.
4. Selection and referral bias: The study population comprised dogs tested, because attending veterinarians suspected tick-borne disease. This clinically selected convenience sample is enriched for exposed and potentially infected animals and is, therefore, not representative of the general dog population. Accordingly, the reported proportions should be interpreted as seropositivity estimates within a diagnostic population rather than as true population prevalence [7, 44].
5. Uneven geographical sampling and sparse strata: A large proportion of samples originated from a small number of districts. Sparse strata (e.g., Setúbal Peninsula) yield unstable estimates and wide CI, limiting fine-scale spatial inference [37].
6. Unmeasured confounding by preventive regimes and management: The multivariable models showed only moderate discriminative ability (AUC ~ 0.70), suggesting that meaningful explanatory variables were not captured (e.g., acaricide/insecticide use, housing, travel, tick infestation history, socio-economic context). This is consistent with broader CVBD epidemiology: preventative regimens can markedly influence exposure risk, and omission of such variables can reduce model performance and obscure causal structure [21, 38].

## Recommendations for future research

Future Portuguese surveillance would benefit from (i) parallel molecular testing (PCR) and/or blood smear review for clinically compatible cases to support species-level attribution and distinguish active infection from past exposure [3, 17, 20, 24]; (ii) integrated tick surveillance to map vector abundance, lineage structure, and pathogen carriage across ecological gradients [4, 18, 23, 28]; and (iii) prospective, standardised data capture on preventative regimens and dog lifestyle, enabling robust modelling of modifiable risk factors and targeted interventions [4, 8, 21, 23]. Given the One Health relevance of tick-borne pathogen circulation and the role of dogs as sentinels, cross-sector surveillance frameworks remain especially timely in Portugal [4, 23].

## Conclusions

In this nationwide, laboratory-based, long-term retrospective study of clinically suspected dogs tested in Portugal between 2015 and 2025 (*n* = 2960), serological exposure to *Babesia* spp. was more frequent than exposure to *E. canis*. Based on definitive ELISA outcomes, 337/2,818 dogs (12.0%) were seropositive for *Babesia* spp. and 116/2,872 (4.0%) for *E. canis*, while co-seropositivity was uncommon (32/2,960; 1.1%). Seropositivity to one agent was associated with seropositivity to the other, consistent with overlapping tick-borne exposure pathways. Regional variation was observed for *E. canis*, with higher exposure pressure in southern Portugal, whereas no statistically significant regional association was detected for *Babesia* spp. in this dataset. As IgG serology reflects cumulative exposure rather than active infection, and as the study population was clinically selected, these findings should be interpreted as reflecting patterns of serological exposure within a diagnostic population rather than prevalence in the broader canine population. Nevertheless, these findings support geographically targeted, risk-based tick control and sustained One Health surveillance, with dogs serving as practical biosentinels of local tick-borne pathogen exposure of relevance to both veterinary and public health.

## Data Availability

Data supporting the main conclusions of this study are included in the manuscript.

## Abbreviations

aOR: Adjusted odds ratio
AUC: Area under the (ROC) curve
CI: Confidence interval
CME: Canine monocytic ehrlichiosis
CVBD: Canine vector-borne diseases
ELISA: Enzyme-linked immunosorbent assay
IgG: Immunoglobulin G
MSF: Mediterranean spotted fever
NUTS: Nomenclature of Territorial Units for Statistics
OR: Odds ratio
PCR: Polymerase chain reaction
ROC: Receiver operating characteristic
s.l.: Sensu lato
